# Molecular mechanisms of an antimicrobial peptide piscidin (Lc-pis) in a parasitic protozoan, *Cryptocaryon irritans*

**DOI:** 10.1186/s12864-018-4565-5

**Published:** 2018-03-12

**Authors:** Ruanni Chen, Yong Mao, Jun Wang, Min Liu, Ying Qiao, Libing Zheng, Yongquan Su, Qiaozhen Ke, Weiqiang Zheng

**Affiliations:** 1 State Key Laboratory of Marine Environmental Science, College of Ocean and Earth Sciences, Xiamen University, Xiamen, Fujian 361005 China; 2 State Key Laboratory of Large Yellow Croaker Breeding, Fujian Fuding Seagull Fishing Food Co., Ltd, Ningde, Fujian 352103 China

**Keywords:** *Cryptocaryon irritans*, Fish disease, Piscidin, Transcriptome, Molecular mechanism

## Abstract

**Background:**

*Cryptocaryon irritans* is an obligate parasitic ciliate protozoan that can infect various commercially important mariculture fish species and cause high lethality and economic loss. Current methods of controlling this parasite with chemicals or antibiotics are widely considered to be environmentally harmful. Piscidins with broad spectrum antibacterial, antifungal and antiviral activities were found to have potent activity against *C. irritans.* Little, however, has been understood about the killing mechanisms of piscidins in parasites.

**Results:**

In total, 57.12, 50.44, 55.86 and 47.87 million raw reads were generated from untreated theront and trophont, and piscidin (Lc-pis) treated theront and trophont libraries, respectively. After de novo assembly, 966,609 unigenes were generated with an average length of 420 bp: among these, 618,629 unigenes showed identity with sequences in one or more databases, with some showing to be significantly manipulated by Lc-pis treatment. The species classification showed that more than 25.8% unigenes from trophonts were homologous to the large yellow croaker (*Larimichthys crocea*) and less than 3.8% unigenes from theronts were matched. The homologous unigenes demonstrated that the tissue from host could exist in trophonts and might be transported to parasite via vesicular transports. Our analysis showed that regulatory transcripts were involved in vesicular trafficking. Among transcripts induced by Lc-pis, most genes up-regulated in treated and untreated theronts were involved in cell migration and apoptosis related pathways. Few transcripts were found to be down-regulated in treated and untreated trophonts related to cell structure and migration after treatment.

**Conclusions:**

This is the first transcriptome analysis of *C. irritans* exposed to Lc-pis, which enhanced the genomic resources and provided novel insights into molecular mechanisms of ciliates treated by cationic antimicrobial peptide. Our comprehensive transcriptome analysis can facilitate the identification of potential drug targets and vaccines candidates for controlling this devastating fish pathogen.

**Electronic supplementary material:**

The online version of this article (10.1186/s12864-018-4565-5) contains supplementary material, which is available to authorized users.

## Background

*Cryptocaryon irritans*, the pathogen of the marine white spot disease (cryptocaryonosis), is an obligate parasitic ciliate protozoan that undergoes four developmental stages, including the infective stage (theront), the feeding stage (trophont), the pre-reproductive stage (protomont) and the reproductive stage (tomont) [1, 2]. Cryptocaryonosis has had a significant negative impact on the mariculture industry worldwide, resulting in huge economic losses [2]. In southern China, *C. irritans* has afflicted mariculture fish species including groupers (e.g. *Epinephelus* spp.), sea breams (Sparidae) and large yellow croaker (*Larimichthys crocea*), which can result in up to 75% mortality [3]. The large yellow croaker has been widely cultured since the 1990s, mainly in floating cages; in terms of the estimated national marine fish culture production (volume) at species level the croaker is currently at the first rank [4]. However, this industry has suffered significant economic losses due to cryptocaryonosis [5]. Current methods of controlling this parasite with chemicals or antibiotics are widely considered to be environmentally harmful.

Antimicrobial polypeptides (AMPs) have been receiving considerable attention as effective and environmentally friendly commercial therapeutics against white spot disease. Piscidins, one of the most common AMPs families, exhibit potent broad spectrum antibacterial, antifungal and antiviral activities [6]. The biological activity of different piscidins isolated from fishes have been tested against gram-positive and gram-negative bacteria (prokaryotes) as well as protozoans (eukaryotes) [7, 8]. Previous studies showed that piscidins were also active against several fish ectoparasites in addition to *C. irritans*, including *Amyloodinium ocellatum dinospore*, *Ichthyophthirius multifiliis* and *Trichodina* spp. [9]. The killing mechanism of AMPs are known to bind lipopolysaccharide, cell wall components and DNA [10, 11]. Many studies focused on the bacterial phospholipid membrane as the main target of these peptides. The barrel-stave model as a possible mechanism of action of piscidins in bacteria has shown to be similar with that in fungi [12–14]. Only in cancer cells, peptides were reported to induce membrane destruction, intracellular calcium mechanism and apoptosis triggered by binding to the mitochondrial membrane [15, 16]. Few studies, however, attempted to undertake any molecular analysis about killing mechanism of piscidins in parasites.

According to our previous study, a gill-expressed piscidin identified in the large yellow croaker (Lc-pis) was lethal to *C. irritans* [17]*.* Killing the detached trophont or the infective theront can stop the reproductive cycle and prevent spread of this disease. RNA sequencing (RNA-Seq) is a powerful approach for whole-transcriptome analysis and can provide an extremely precise measurement in model and non-model organisms [18, 19]. It has been broadly applied in studies of the molecular mechanisms including stress resistance, models of development and the immune defenses [20, 21]. Transcriptomic analyses of *C. irritans* have been conducted on different developmental stages and transformations after low temperature treatments [22–25]. We analyzed transcriptome variances among different developmental stages and compared the changes in transcriptomes after treated by Lc-pis. Furthermore, we documented the evidence for the modulation of the *C. irritans* transcriptome when exposed to the antiparasitic Lc-pis and revealed different responses in different developmental stages of *C. irritans*.

## Methods

### Test peptide

Lc-pis (IWGLIAHGVGHVGRLIHGLIRG) with a purity of > 98% was synthesized (China Peptides Co. Ltd., Shanghai, China). After synthesis, reversed phase high-performance liquid chromatography (RP-HPLC) was used to purify Lc-pis. The chromatographic condition was: 0.1% trifluoroacetic acid (TFA) in water as Buffer 1 while 0.1% TFA in acetonitrile as Buffer 2, and the chromatographic condition was 15%–60% Buffer 2 on a Kromasil 100–5 C18 (4.6 mm × 250 mm, 5 μm) column at the flow rate of 0.2 mL/min for 15 min at 35 °C. Lc-pis was detected by its absorbance at 220 nm. After purification, an API-150EX mass spectrometer with the ion source of electrospray ionization was used to detect and identify the peptide. Sterile deionized water was used to dilute Lc-pis prior to *C. irritans* treatment.

### Sample preparation and treatment

Forty juveniles of the large yellow croaker (weight 78.5 ± 9.1 g, total length 19.4 ± 0.9 cm) were obtained from a commercial fish farm at Gulf of Sandu, Fujian Province, China in October, 2015. Then the fish were infected with a non-lethal concentration of *C. irritans* (about 8000 theronts/fish) in 800 L of seawater. The collection method for *C. irritans* was described previously [26]. The water was oxygenated continuously and replaced every three days, and the salinity, water temperature and photoperiod were maintained at 23–25‰, 23 ± 1 °C and 12 L: 12D, respectively. The trophont were gently scraped from skin and gills 3 days after infection and washed with sterilized seawater more than three times to clear fish tissues. Within four days, mature trophonts were obtained using standard procedures [27]. Theronts incubated within 2 h in sterilized seawater were filtered (pore size 0.22 μm; GSWP04700, Millipore Corp.) and collected. In order to understand the genetic basis for differences after treated by Lc-pis, four cDNA libraries isolated from trophonts (control: ZYT-c and treatment: ZYT-t) and theronts (control: YC-c and treatment: YC-t) were established. The four treatment groups were divided. Theronts and trophonts were treated with Lc-pis at a final concentration of 1.5 μM for 90 min in order to maintain the RNA quality of *C. irritans* population. Theronts and trophonts without Lc-pis treatment were used as controls. Each sample was performed in triplicate. *Cryptocaryon irritans* were immediately cryopreserved in liquid nitrogen until RNA isolation. Animal treatment in this study was performed in strict accordance with the recommendations of Animal Care Quality Assurance in China.

### Library construction and Illumina sequencing

Total RNA was extracted using MiniBEST Universal RNA Extraction Kit (TaKaRa 9767) according to the manufacturer’s instructions. Triplicate RNA were merged into one sample. RNA degradation and contamination was monitored on 1% agarose gels. RNA concentration and integrity were measured using a Qubit RNA Assay Kit in Qubit2.0 Flurometer (Life Technologies, CA, USA) and RNA Nano 6000 Assay Kit of the Agilent Bioanalyzer 2100 system (Agilent Technologies, CA, USA). The four mRNA-seq libraries were performed at Novogene Co., Ltd. (Beijing, China) with a NEBNext Ultra RNA Library Prep Kit for Illumina (New England Biolabs, USA) following the manufacturer’s recommendations. mRNA was purified from total RNA using poly-T oligo-attached magnetic beads. First strand cDNA was synthesized using random hexamer primers and M-MLV Reverse Transcriptase (RNaseH-). Second strand cDNA synthesis was subsequently performed using DNA Polymerase I and RNase H. DNA fragments were treated for end-repairing, adenylation of 3′ ends and ligation of adaptors. The library fragments were purified with AMPure XP system (Beckman Coulter, CA, USA) to preferentially select cDNA fragments of 150~ 200 bp in length, and suitable fragments were enriched by PCR amplification.

### Assembly of sequencing and gene annotation

The clustering of the index-coded samples was performed on a cBot Cluster Generation System using TruSeq PE Cluster Kit v3-cBot-HS (Illumia) according to the manufacturer’s instructions. After cluster generation, the library preparations were sequenced on an Illumina Hiseq platform and 2 × 125 bp paired-end reads were generated. Raw sequences were deposited to NCBI Short Read Archive (SRA) database (http://www.ncbi.nlm.nih.gov/Traces/sra/). After removing adaptor sequences, ambiguous ‘N’ nucleotides (with the ratio of ‘N’ to be more than 10%) and low quality sequences (with quality score to be less than 5), the remaining clean reads were assembled using Trinity for transcriptome assembly without reference genome [19]. The longest transcript of each single gene was selected as a unigene. For annotation analysis, unigenes were BLASTX-searched against seven databases, including the National Center for Biotechnology Information (NCBI) non-redundant protein sequence (Nr) database, non-redundant nucleotide sequence (Nt) database, Protein family (Pfam), Clusters of Orthologous Groups (KOG/COG), Gene ontology (GO), Kyoto Encyclopedia of Genes and Genomes (KEGG) Orthology (KO) database, and the Swiss-Prot, using a cut-off E-value of 10–5. Differentially expressed genes (DEGs) between untreated therents and trophonts were identified with DEGseq analysis on adjusted read count data. To identify unigenes involved in *C. irritans* responsing to antiparasitic peptide treatment, pairwise comparisons for differential expression analysis were conducted among theront treatment and its control group, and trophont treatment and its control group. Unigenes were annotated based on BLASTX results, and the best alignments were used for downstream analyses. KEGG database were used to predict the functions of unigenes [28, 29].

### Experimental validation of transcription levels

RNAs from four treatment groups (theronts and trophonts treated by Lc-pis; theronts and trophonts without Lc-pis treatment) were extracted with method mentioned above. Replicate samples were run for real time quantitative-PCR (RT-qPCR) validation of Illumina sequencing. First-strand cDNA was synthesized using the PrimeScript RT reagent Kit with gDNA Eraser (TaKaRa DRR034A) according to the manufacturer’s protocol. Specific primer for five genes (heat shock protein 90, GTP-binding protein, serine/threonine kinase, serum/glucocorticoid regulated kinase, Rab 5) were designed (Additional file 1). PCR products were purified and cloned using pMD™19-T Vector (TaKaRa, Japan). The plasmids carrying target genes were extracted and purified using AxyPrep Plasmid DNA Miniprep Kit (Axygen, America). Calibration curve was generated for qPCR using tenfold serial dilution of the plasmid. Quantitative PCR was performed using ABI Prism 7500 RT PCR machine with SYBR Premix DimerEraser (TaKaRa RR091A). Thermo cycling condition was 95 °C for 30 s, followed by 40 cycles at 95 °C for 3 s, 60 °C for 30 s and 72 °C for 30 s. For direct comparison with the results in transcriptome, qPCR results were converted to log2-fold changes. A Pearson’s correlation test was calculated using GraphPad Prism 5.0.

### Scanning electron microscopy (SEM)

Sample preparation for SEM was conducted according to previous methods [30]. Collected samples were added to 2.5% glutaraldehyde at 4 °C for 24 h. Theronts were fixed in a 6:1 mixture of saturated HgCl_2_ and 1% O_S_O_4_ at 4 °C for 10 min. All solutions were diluted in sodium cacodylate buffer (pH 7.2). After washing, samples were dehydrated in a graded ethanol series, critical-point dried, placed on aluminium stubs, and sputter coated with platinum. Prepared samples were observed with a Hitachi S-4800 SEM.

### Data availability

The sequence data of the present study have been deposited into SRA (the accession numbers: SRX2417025, SRX2417144, SRX2417145, and SRX2417163).

## Results

### Effect of Lc-pis on *C. irritans*

**Fig. 1:**
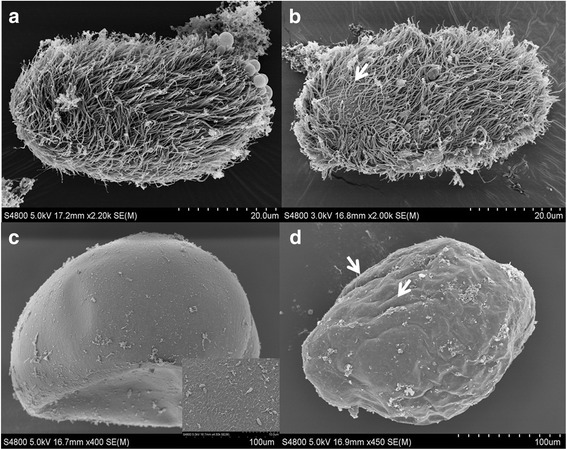
Images of microscopic structure of the theront and trophont under scanning electron microscopy (SEM). Surface of the theronts for control group (**a**) and for 1.5 μM Lc-pis treated (**b**), arrow indicates the region without cilium; the trophonts for control group (**c**) and for Lc-pis treated (**d**), arrows mark the uneven surface

Given our goal of investigating the ways *C. irritans* are effected*,* it is important to explore both structures and genes regulated by Lc-pis. The light microscopy results showed that Lc-pis quickly induced the membrane and cytoplasmic leakage rupture of theront and trophont ruptur in cells (data not shown). Under SEM, theronts lost part of cilium and changed their shapes from pyriform to round. The uneven surface of treated trophonts could be ascribed to specific or non-specific interaction of Lc-pis with the membrane (Fig. 1). Only few parts of trophonts post stimulus had cytoplasmic leakage.

### Transcriptome sequencing and assembly

In total, 57.12, 50.44, 55.86 and 47.87 million raw reads were generated from untreated theront and trophont, and Lc-pis treated theront and trophont libraries, respectively (Table 1). A reference transcriptome was obtained from all raw reads from the four cDNA libraries. Clean reads were acquired with a Q20 percentage of over 96.48%, which were assembled into 966,609 transcripts with a mean length of 420 bp and an N50 length of 463 bp (Table 2). An overview of the sequencing results was summarized in Additional file 2.

**Table 1:** Quality parameters of Illumina transcriptome sequencing of *Cryptocaryon irritans*

Sample	Raw Reads	Clean Reads	Clean Bases	Error (%)	Q20 (%)	Q30 (%)	GC Content (%)
YC_c	57,123,566	55,352,132	8.30G	0.02	96.48	92.01	36.12
YC_t	55,860,980	53,930,928	8.09G	0.02	96.50	91.86	40.72
ZYT_c	50,439,260	48,942,064	7.34G	0.02	96.64	92.12	37.86
ZYT_t	47,869,336	46,800,222	7.02G	0.01	96.97	92.72	35.35

**Table 2:** Length distribution of assembled transcripts and unigenes

	Min Length	Mean Length	Median Length	Max Length	N50	N90	Total Nucleotides
Transcripts	201	420	267	14,764	463	220	406,360,243
Unigenes	201	390	262	14,764	390	217	344,660,742

### Homology analysis and gene functional annotation

**Fig. 2:**
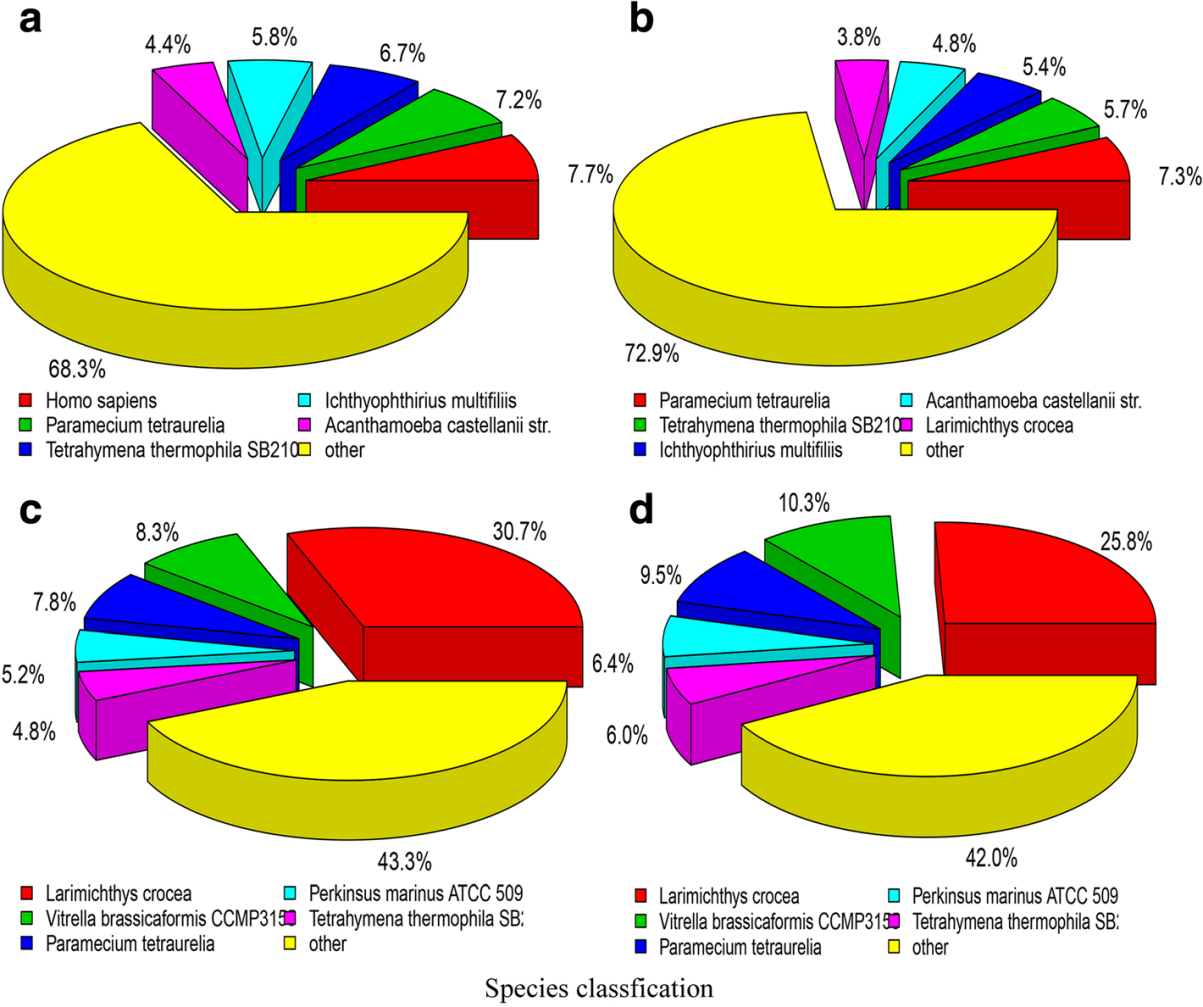
Species distribution of gene annotations in transcriptional profile. BLASTx top-hit species distribution of gene annotations in transcriptional profile of theronts (**a**), treated theronts (**b**), trophonts (**c**) and treated trophonts (**d**) of Cryptocaryon irritans

In total, 884,341 non-redundant unigenes were found, among these, 214,488 matched to known proteins in the Nr database, 416,413 matched to putative homologues in the Nt database and only 33,287 unigenes matched to KO database. In total, 618,629 unigenes (69.95%) showed identity with sequences in one or more databases (Additional file 3). The e-value distribution showed that 24.4% of the annotated unigenes had strong homology (e-value <1e-45), whereas 33.8% of the unigenes had low homology (1e-15 < e-value<1e-5). The species classification showed that more than 25.8% unigenes of trophonts were homologous to the large yellow croaker but less than 3.8% unigenes of theronts were matched. Sequence homologues for over 68% unigenes of the predicted peptides of theronts and about 42% of trophonts were found to be associated with others including bacteria. Unigenes associated with many kinds of protozoa showed that the unigenes reveal high conservation between protozoa. Homology analysis is shown in pie charts in Fig. 2.

**Fig. 3:**
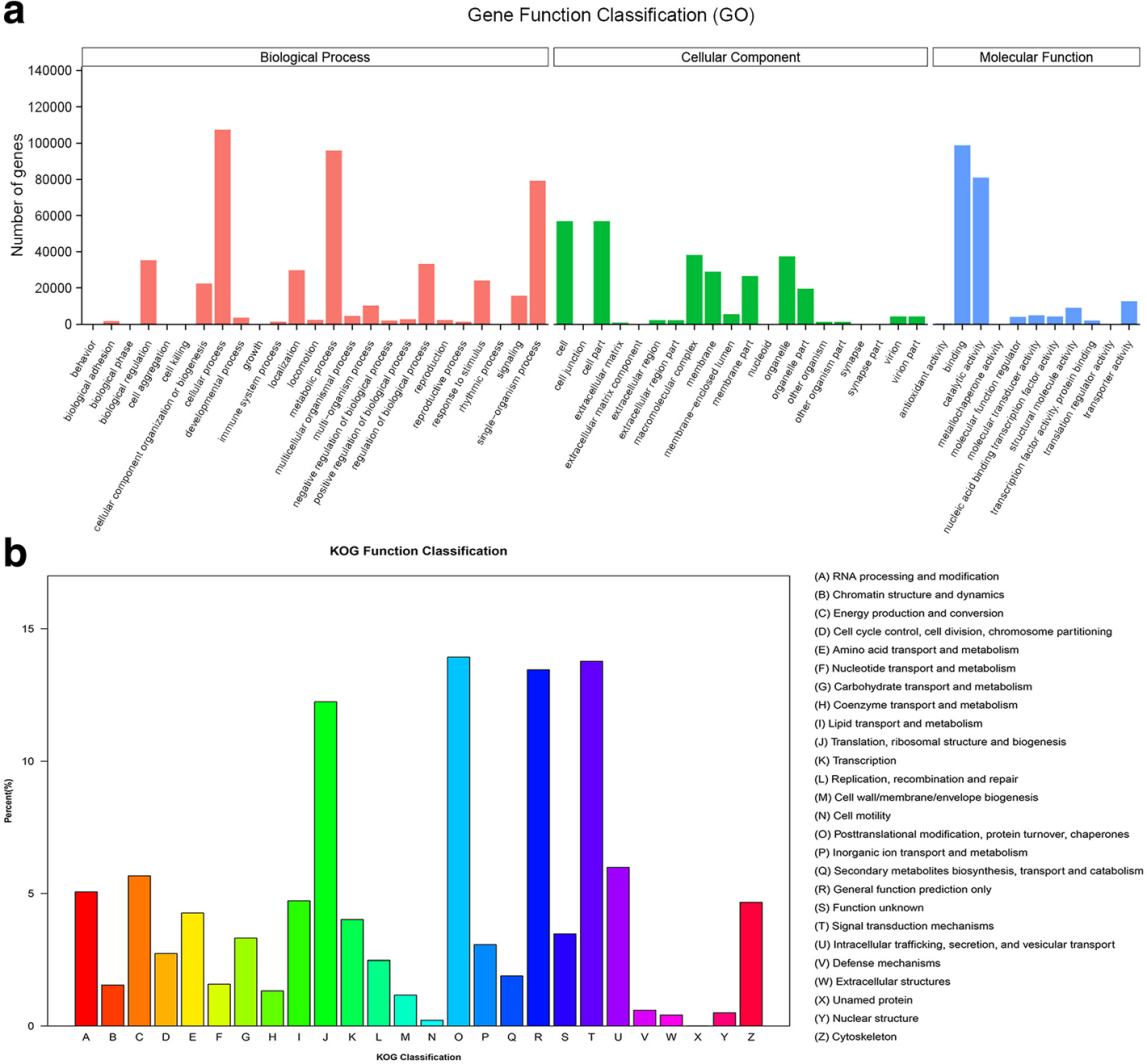
GO annotation and histogram presenting clusters of orthologous groups (KOG) classifications. Of 884,341 unigenes, 114,478 sequences were assigned to 26 KOG classifications. The GO results were summarized in three main GO categories: biological process, cellular component and molecular function. The x-axis indicates the subcategories, and the y-axis indicates the numbers related to the total number of GO terms present

In total, 201,906 DEGs were classified into three major functional categories (biological process, cellular component and molecular function) and 55 subcategories (Fig. 3). Additional file 4 represents GO enrichment analysis of DEGs from above three groups [31]. With the KOG classification, 128,322 matched unigenes were clustered into 26 categories. Among these categories, the largest group was group O-posttranslational modification, protein turnover and chaperones (15,942; 12.42%), followed by group T-signal transduction mechanisms (15,763; 12.28%), while only 8 unigenes were classified as unnamed protein (Fig. 3).

### Hierarchical clustering of DEGs among transcriptomes

**Fig. 4:**
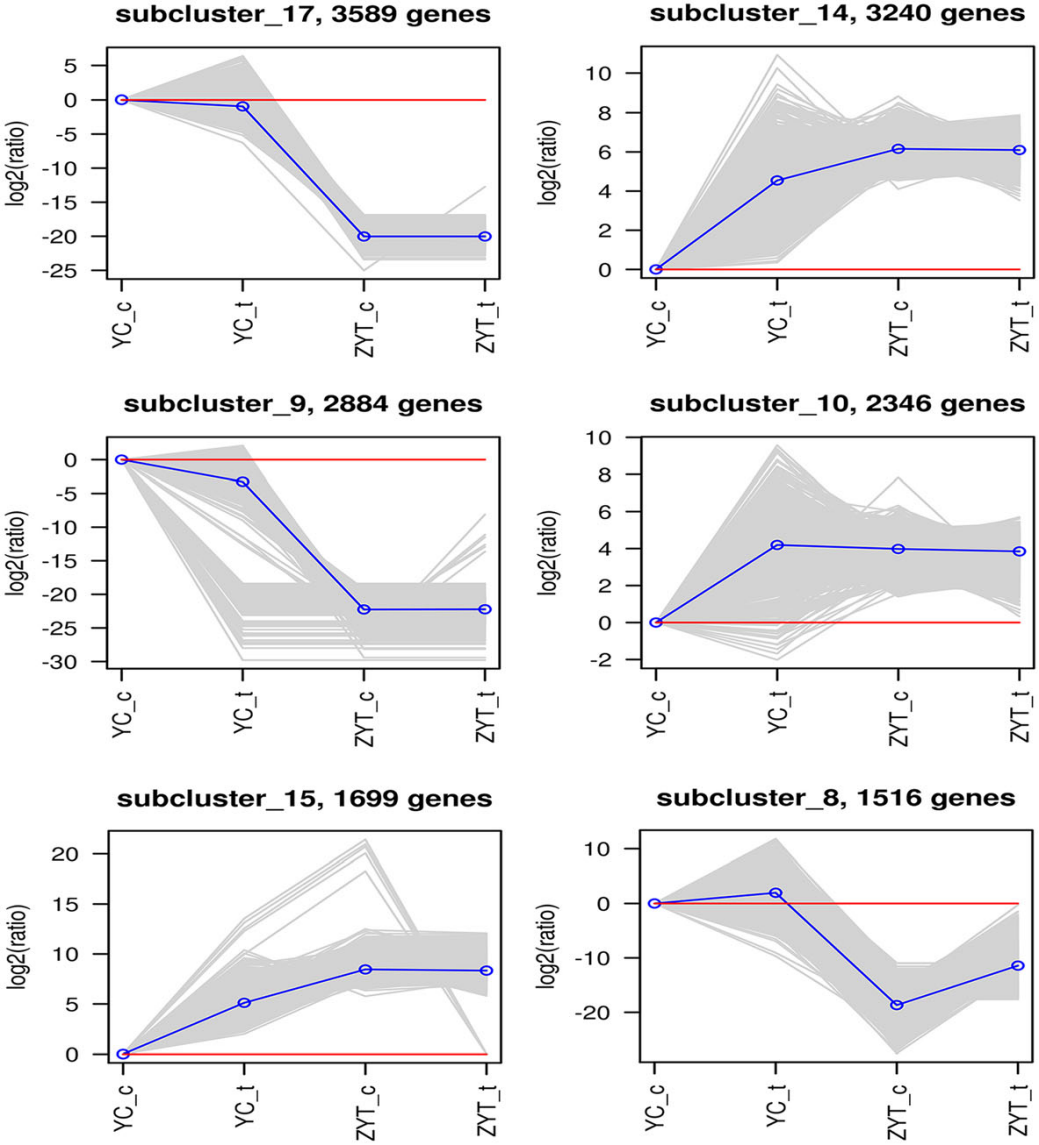
The top six clusters determined by K-means clustering, including 15,274 DEGs (68.8% of all genes). The x-axis indicates different group. The y-axis indicates the log2 (ratio) of gene expression. Each grey row represents the relative expression of DEGs in a cluster. The blue line represents the average value of all members. The red line denotes reference, the line above the red line represents up-regulation, and the line below the red line represents down-regulation. The number of DEGs within a cluster is shown after that subcluster

Expression levels of treatment and control groups were divided into 18 categories based on K-means clustering. Detailed expression profile clusters were shown in Additional file 5. The largest subcluster 17 contained 3589 DEGs with decreasing expression levels from the theront to trophont; the same trend was also observed in subclusters 2 (2664 DEGs), 8 (1516 DEGs), 9 (2884 DEGs), 11 (759 DEGs), 12 (1022 DEGs) and 18 (559 DEGs). The expression patterns in most subclusters (e.g. 1 (1506 DEGs), 3 (508 DEGs), 4 (900 DEGs) and 5 (467 DEGs)) showed that DEGs regulated positively in theronts after Lc-pis treatment while DEGs were down-regulated in treated trophonts (Fig. 4).

### Stage-specific gene expression of theronts and trophonts

Functions of the 8751 transcripts were assigned to GO classes with 4626 functional terms. The profile between YC-c and ZYT-c is shown in Additional file 6. Of 17,373 unigenes that were differentially expressed, 8575 were up-regulated and 8798 down-regulated. The pathways with most genes were the ribosome (ko 03010), vibrio cholerae infection (ko 05110), prion diseases (ko 05020), pathogenic *Escherichia coli* infection (ko 05130), cocaine addiction (ko 05030) and phagosome (ko 04145) (Table 3). Among them, the phagosome pathway contained 83 DEGs, including actin-associated protein coronin, dynein, *Rab5* and major histocompatibility complex class I (*MHC I*). Genes such as *Rab11b*, *Rab7a*, dynamin mapping to endocytosis pathway (ko 04144) and extracellular signal-regulated kinase1/2 (*ERK 1/2*), cofilin, gelsolin and actin related proteins 2/3(*Arps 2/3*) mapping to Fc gamma receptor-mediated phagocytosis pathway (ko 04666) were also related to vesicular trafficking.

**Table 3:** The top 20 most up-regulated and down-regulated genes between theronts and trophonts. The rich factor refers to the ratio of the number of differentially expressed genes in the pathway and the number of all genes in the pathway

Pathway_term	Rich_factor	q-value	Gene_Number
Ribosome	0.142451	4.84E-13	351
Vibrio cholerae infection	0.143921	0.075845	58
Prion diseases	0.170000	0.075845	34
Pathogenic *Escherichia coli* infection	0.165877	0.075845	35
Cocaine addiction	0.194444	0.097259	21
Phagosome	0.127301	0.097259	83
mTOR signaling pathway	0.134518	0.191181	53
Oxidative phosphorylation	0.116246	0.521090	83
Parkinson”‘“s disease	0.115033	0.521090	88
Ribosome biogenesis in eukaryotes	0.119772	0.561419	63
Steroid biosynthesis	0.172840	0.634712	14
Non-alcoholic fatty liver disease (NAFLD)	0.115702	0.642030	70
Hedgehog signaling pathway	0.135266	0.696605	28
Ovarian steroidogenesis	0.153846	0.792778	16
Vasopressin-regulated water reabsorption	0.125000	0.792778	36
Thyroid hormone synthesis	0.122517	0.794620	37
Protein export	0.138889	0.794620	20
Plant-pathogen interaction	0.118457	0.794620	43
Apoptosis	0.123134	0.794620	33
Toxoplasmosis	0.113131	0.794620	56

### Comparative gene expression of response to Lc-pis treatment

The GO profiles showed that 8751 transcripts with significantly differing transcript levels occurred in the theront group but not found in the trophont group. The unigenes were mapped to the reference pathways recorded in the KEGG database. In the theront group, the ribosome related pathways were also the most enriched when compared to the stage of trophonts (Additional file 7). Transcripts such as tenascin and histones were highly regulated in group treated with piscidins. Beyond that, a number of DEGs were involved in different pathways causing cell death (i.e. apoptosis, antigen processing and presentation, lysosome, mammalian target of rapamycin and phosphatidylinositide 3-kinase signaling pathway) after being treated with Lc-pis.

**Fig. 5:**
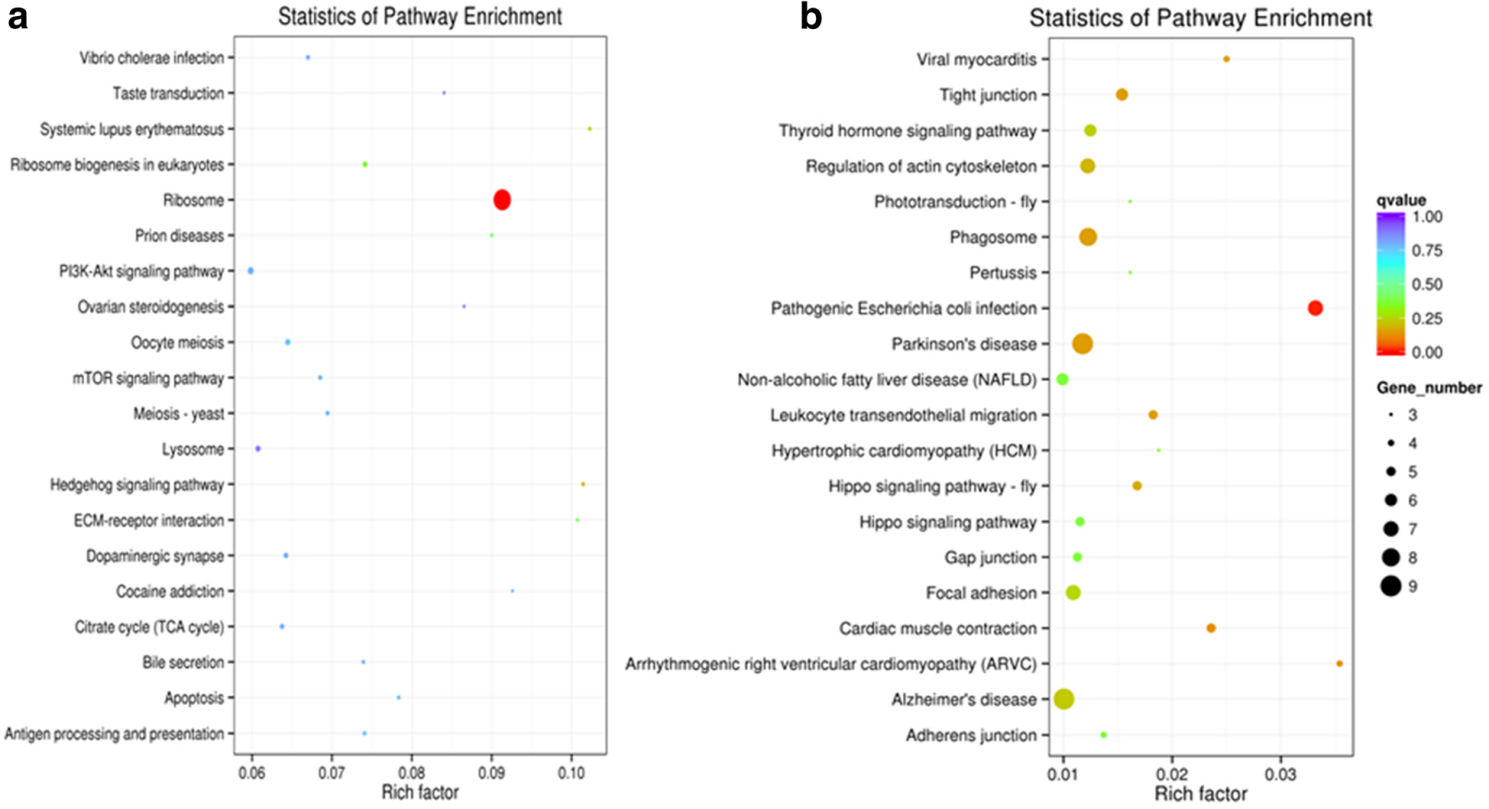
KEGG pathway enrichment analysis of the differently expressed genes between the theront group (**a**) and trophont group (**b**)

Only 283 transcripts with significantly differing transcript levels were found in the trophont group; among these, 215 DEGs were down-regulated. DEGs in the trophont group were classified into protein polymerization (GO:0051258), generation of precursor metabolites and energy (GO:0006091), chemokine activity (GO:0008009), chemokine receptor binding (GO:0042379), receptor binding (GO:0005102), actin monomer binding (GO:0003785). All function terms above were involved in the theront group (Additional file 7); however, only few DEGs, such as alpha-tubulin, beta tubulin and cytochrome c oxidase subunit were involved in the trophont group and transcripts with significant alteration were of low amplitude. Other terms including leukocyte transendothelial migration, tight junction, phagosome, regulation of actin cytoskeleton, focal adhesion, adherens junction and gap junction were associated with membrane or cytoskeleton structure, while others were associated with stimulus induced or apoptosis related pathways (Fig. 5).

### Corroboration of illumina sequencing approach by RT qPCR

Expression values determined by the RT-qPCR analysis of five genes were found to be comparable to the RNA-seq approach (Additional file 8). The values obtained by RT qPCR and RNA-seq were highly similar and correlated with statistical significance (*r* = 0.523, *p* = 0.060; Additional file 8).

## Discussion

Large amounts of antibiotics and chemicals are used to prevent pathogens in fish farms, which can induce the antibiotic resistance and damage the surrounding aquatic environment [32]. New approaches to combat infectious diseases need to be considered. AMPs, known as nature’s defenders, have been identified in almost all living organisms. [6, 33]. One group of AMPs is the piscidin family, which has recently been found to have potent antiparasitic activities [9, 34–36]. In our previous study, Lc-pis was proved to have strong antiparasitic activities against the parasite *C. irritans* [17], which affects many teleosts and was responsible for significant economic losses to the aquaculture industry [1, 37]. The minimum lethal oxygen concentration of Lc-pis for theronts is 1.5 μM after 90 min (less than 60 min for 2 μM and non-efficent for 1 μM) while tomont, with a thick cyst wall, were non-susceptible at such low concentration (data not show). Here, we explored, for the first time, the cytoskeleton structure of *C. irritans* after Lc-pis treatment and identified putative mechanisms using transcriptome data.

The majority of unigenes were shorter than 500 bp, and this phenomenon existed in some nonmodel organisms including the transcriptomes of *C. irritans* from tomont stage [22, 38, 39]. These short unigenes might come from assembly errors, fragmented transcripts of low expressed genes, as well as noncoding RNA. Of the short unigenes, 69.95% were annotated in at least one database. Part of the annotated unigenes were matched to the large yellow croaker in the trophont group, while few fish genes were found in the theront group. This indicates that the trophonts were fed with host cells, which seemed to proceed throughout the trophont stage. *Cryptocaryon irritans* cannot be cultured without host, and contamination by host RNA cannot be completely eliminated when preparing samples. This was also observed in *C. irritans* and *Ichthyophthirius multifiliis* [20, 23]. In addition, previous researches showed parasite genes have revealed high degrees of identity between the nucleotide sequences of parasite and mammalian genes [40, 41]. These genes might help parasites escape host immunosurveillance. Other unigenes revealed high conservation between protozoa which could be conjectured to participate in similar mechanisms in protozoa like basic cellular mechanisms.

As *C. irritans* progresses through its lifecycle, it must adapt to different environments with differing energy requirements and this is reflected in differential gene expressions. A previous transcriptional analysis of *C. irritans* demonstrated that metabolic enzymes were highly expressed in the trophont stage [23]. Cytoskeletal proteins such as actin and tubulins were discovered to be persistent throughout the whole life cycle of *C. irritans* in an immunoproteomics analysis, and studies in mammals indicated they could result to the interplay of signaling pathways and cytoskeletal dynamics [24, 42]. Rab family proteins and dynein were both proved to take part in regulating exocytosis and endocytosis [43]. As many protozoa are incapable of synthesizing purines de novo, enzymes involved in purine salvage are of particular importance [44–46]. ATP synthases highly expressing in the trophont stage were observed in our study. For parasites, the infection stage is the only stage to get nutrition from their hosts. Recently, the ingestion of host cells, occurred in the trophont and the early tomont stages, was investigated by using in situ hybridization [47]. The present analysis of theronts and tomonts also demonstrated that metabolic related and cytoskeleton associated genes were enriched especially in vesicular transport pathways. Thus, it was reasonable to hypothesize that phagosome functions occurred prior to the trophont stage and were shed by host tissues and the typical genes demonstrated the mechanism of phagosome formation of endoparasitic ciliates might similar to other eukaryotes.

In the present study, the comparative transcriptome profiling allowed us to clarify the mechanism by which ways the Lc-pis effect on *C. irritans* and provide more potential targets for vaccine development against *C. irritans*. Piscidins have potent antimicrobial activity against a broad spectrum of pathogens in vitro. While knowledge of AMPs mechanisms of action remained unclear, and debate continues as to the relative contributions of proposed pore formation or internal killing strategies [48]. Several studies revealed that amphipathic character seemed to play an essential role in the various mechanisms of membrane permeabilization [48–50]. Piscidins were found to interact with various membrane-mimicking interfaces, suggesting that its interaction with them was not only electrostatically mediated [12].

In the theront group, the ribosome pathway was the first of the most DEGs-enriched pathways. This result was similar to the result of the previous study on *C. irritans* transcriptome [22, 25]. Several cell death pathways were present after stimulation and significant alteration transcripts were found. For example, histones, especially H2A, were associated with pathogenesis in some protozoan parasites [51, 52]. The transcripts annotated for histones (e.g. H2A, H2B, H3 and H4) in present study displayed altered transcription profiles in the theront group treated by Lc-pis or without treatment, which might participate in infection of *C. irritans*. The tenascin family is a large extracellular matrix glycoprotein which transduces and integrates intracellular signals via identifying cell surface receptors. The tenascin-C promoted cell migration upon injury in mice and contributed to migration of fibroblast [53, 54]. However, tenascin was rarely studied in parasites, the roles in *C. irritans* exposed to Lc-pis remains to be demonstrated. Protein kinases are found to play a central role in the cellular signaling pathways and was involved in biological processes as diverse as gene expression, metabolism, apoptosis, and cellular proliferation through phosphorylation of substrate proteins [55]. The schistosome cAMP-dependent protein kinase (PKA) was suggested to be essential for maintaining parasite viability in *Schistosoma mansoni* and was proved to affect flagellar wave in *Leishmania* [56, 57]. A putative PKA was up regulated after stimulation when theronts were observed moving slower than the control group and a large scale of cilia were shed, which indicated that PKA also may take part in the movement of theronts. Further work is required to discover exact killing mechanisms by Lc-pis.

Expression patterns were different between treated trophont and treated theront, observed in K-means analysis. In the present study, DEGs were involved mainly in cell structure and migration after stimulation. Ultrastructural observation of the trophont showed mucus and fibrous materials mixture could be observed surrounding *C. irritans* in vivo [58]. Two tubulin genes, α tubulin and β tubulin (their nucleotide sequences shared over 81% similarity with *Dugesia japonica* and *Lottia gigantean*, respectively), were identified with significant down-regulation. Tubulin is a fundamental constituent of kinetoplastid cytoskeletons, cell division machinery and motile organelle [59]. Studies about tubulin in protozoa suggested that the expression of tubulins varied during the life cycles of *Trypanosoma* and *Leishmania* spp., possibly as the relative demand for these structures fluctuates [60, 61]. The suppression of genes by Lc-pis indicates that this peptide inhibits the trophont from developing into protomont. Until now, research into the response of *C. irritans* to antiparasitics was insufficient. Above all, we noticed that the pore formation might not be the only reason which led to cell death at such a low concentration and the transcriptome analysis after exposed to Lc-pis helped to better understand the killing mechanisms.

## Conclusions

*Cryptocaryon irritans* is an obligate ectoparasitic ciliate pathogen of marine fishes that has caused heavy economic losses in the aquaculture sector. Here, we generated transcriptome sequencings of theronts and trophonts exposed to Lc-pis using a next generation sequencing technology, conducted comprehensive analysis of the stimuli-responsive genes and explored the putative mechanisms of Lc-pis resistance for the first time. The results enlarged the genomic resources of *C. irritans* and the different developmental stage specific transcripts illustrated that vesicular transports were continuously generated throughout the trophont stage. The differences in gene expression pattern between theronts and trophonts might be due to the pellicular swells underneath the pellicle of trophonts. Our transcriptome analyses provide valuable information for further investigation into the molecular mechanisms of ciliates treated by cationic antimicrobial peptides and facilitate the identification of potential drug targets and vaccines candidates for the control of this devastating fish pathogen.

## Additional files

Additional file 1: Primer sets used in RT qPCR validation. (DOCX 14 kb)

Additional file 2: The length distribution of the transcripts (red) and unigenes (blue). (JPEG 447 kb)

Additional file 3: BLAST analysis of non-redundant unigenes against public databases. (XLSX 10 kb)

Additional file 4: Summary of the GO classifications of assembled unigenes. (XLSX 12 kb)

Additional file 5: Eighteen regulatory patterns determined by K-means clustering of all the DEGs expressed in theront and trophont groups of *Cryptocaryon irritans* with Lc-pis treatment. (JPEG 929 kb)

Additional file 6: GO enrichment bar graphs of DEGs between untreated theronts and untreated trophonts of *Cryptocaryon irritans. (JPEG 504 kb)*

Additional file 7: GO classification of the DEGs. A: YC-t vs YC-c, B: ZYT-t vs ZYT-c. (JPEG 641 kb)

Additional file 8: Expression profiles of five genes from four developmental stages of *Cryptocaryon irritans* from RNA-Seq and RT-qPCR. Log2-fold changes from RNA-Seq analysis were highly correlated with log2-fold change values from RT-qPCR. A, B, C, D and E represented heat shock protein 90, GTP-binding protein, serine/threonine kinase, serum/glucocorticoid regulated kinase, Rab 5, respectively. Log2-fold changes are relative to YC c (A, B, C, D and E for ZYT-c/ YC-c; A1, B1, C1, D1 and E1 for vs YC-t/ YC-c). (JPEG 673 kb)

## Abbreviations

AMPs: Antimicrobial polypeptides
DEGs: Differentially expressed genes
HPLC: High-performance liquid chromatography
PKA: cAMP-dependent protein kinase
SEM: Scanning electron microscopy
TFA: Trifluoroacetic acid
