# Supplementary material for: Molecular mechanisms of an antimicrobial peptide piscidin (Lc-pis) in a parasitic protozoan, Cryptocaryon irritans
Source: BMC Genomics. 2018 Mar 12;19:192. doi: 10.1186/s12864-018-4565-5 (PMC6389114; doi:10.1186/s12864-018-4565-5)
Supplement: Supplementary file 1 — Primer sets used in RT qPCR validation. (DOCX 14 kb) [file 12864_2018_4565_MOESM1_ESM.docx]

**Additional file 1:** Primer sets used in RT qPCR validation. A, B, C, D and E represented heat shock protein 90, GTP-binding protein, serine/threonine kinase, serum/glucocorticoid regulated kinase, Rab 5, respectively.

| **Gene** | **Forward primer** | **Reverse primer** |
| --- | --- | --- |
| A | 5′- AGGGTGTTGTAGATTCTGACG-3′ | 5’- TTATCATCTGCCTCCTGCTCT-3′ |
| B | 5’- CTGGAAAGGCTCCTCAAGAC-3’ | 5’- GTCAGAATCTACAACACCCT-3’ |
| C | 5′- TAGATACTACTGGGATTGGGACA-3′ | 5′- ATCAGCAGCTTTGGAATGACC-3′ |
| D | 5’- TATTGCGAGCACCAACTCTG-3’ | 5’- ATGAACTACGCTTTGACTGA-3’ |
| E | 5′- GAAACTCTGACGCTTTGTTAT-3′ | 5′- AGGAGACTCAGGTGTTGGAAA-3′ |
