# Supplementary figures and images for: Molecular mechanisms of an antimicrobial peptide piscidin (Lc-pis) in a parasitic protozoan, Cryptocaryon irritans
Source: BMC Genomics. 2018 Mar 12;19:192. doi: 10.1186/s12864-018-4565-5 (PMC6389114; doi:10.1186/s12864-018-4565-5)

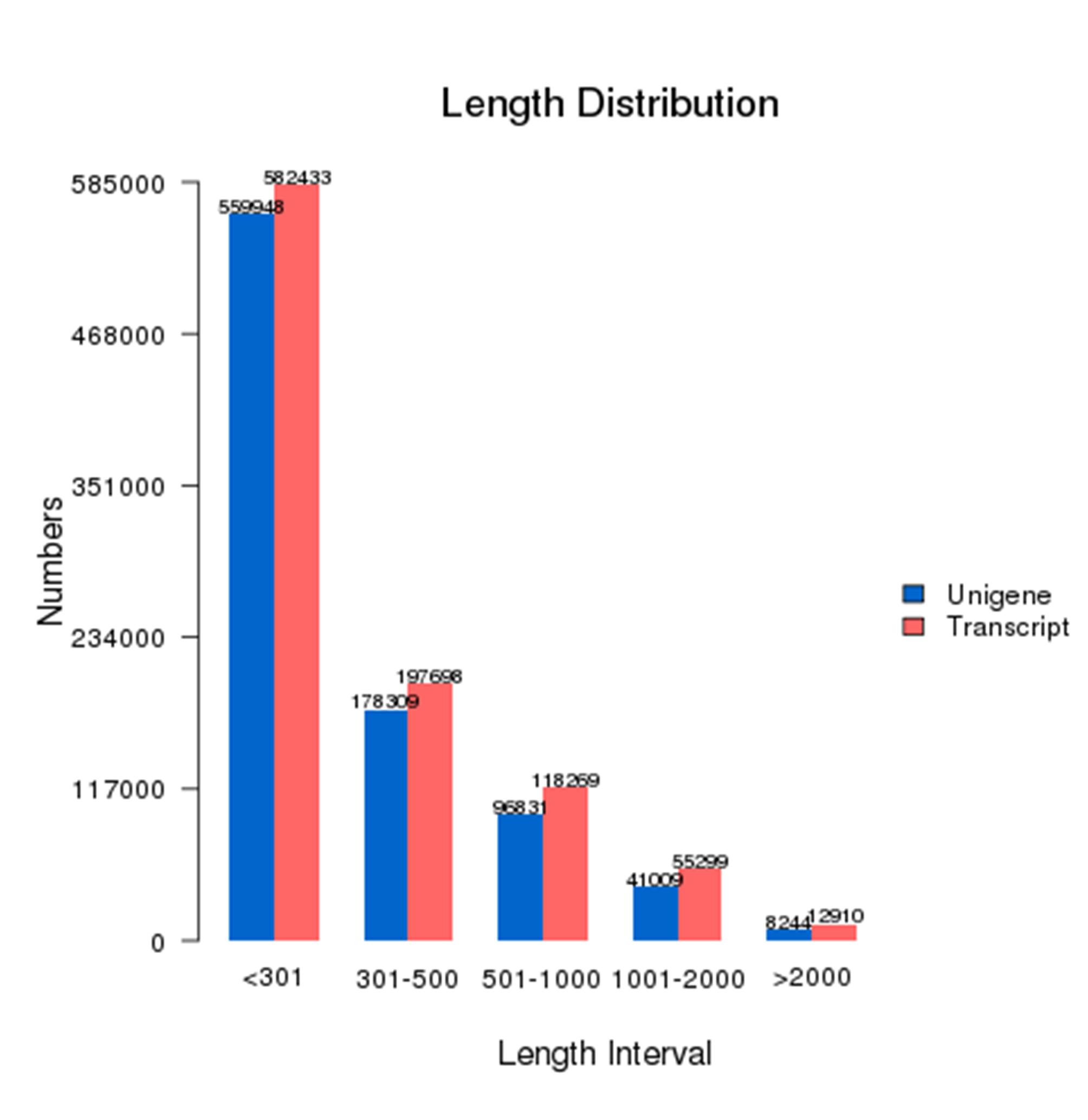

Supplement: Supplementary file 2 — The length distribution of the transcripts (red) and unigenes (blue). (JPEG 447 kb) [file 12864_2018_4565_MOESM2_ESM.jpg]

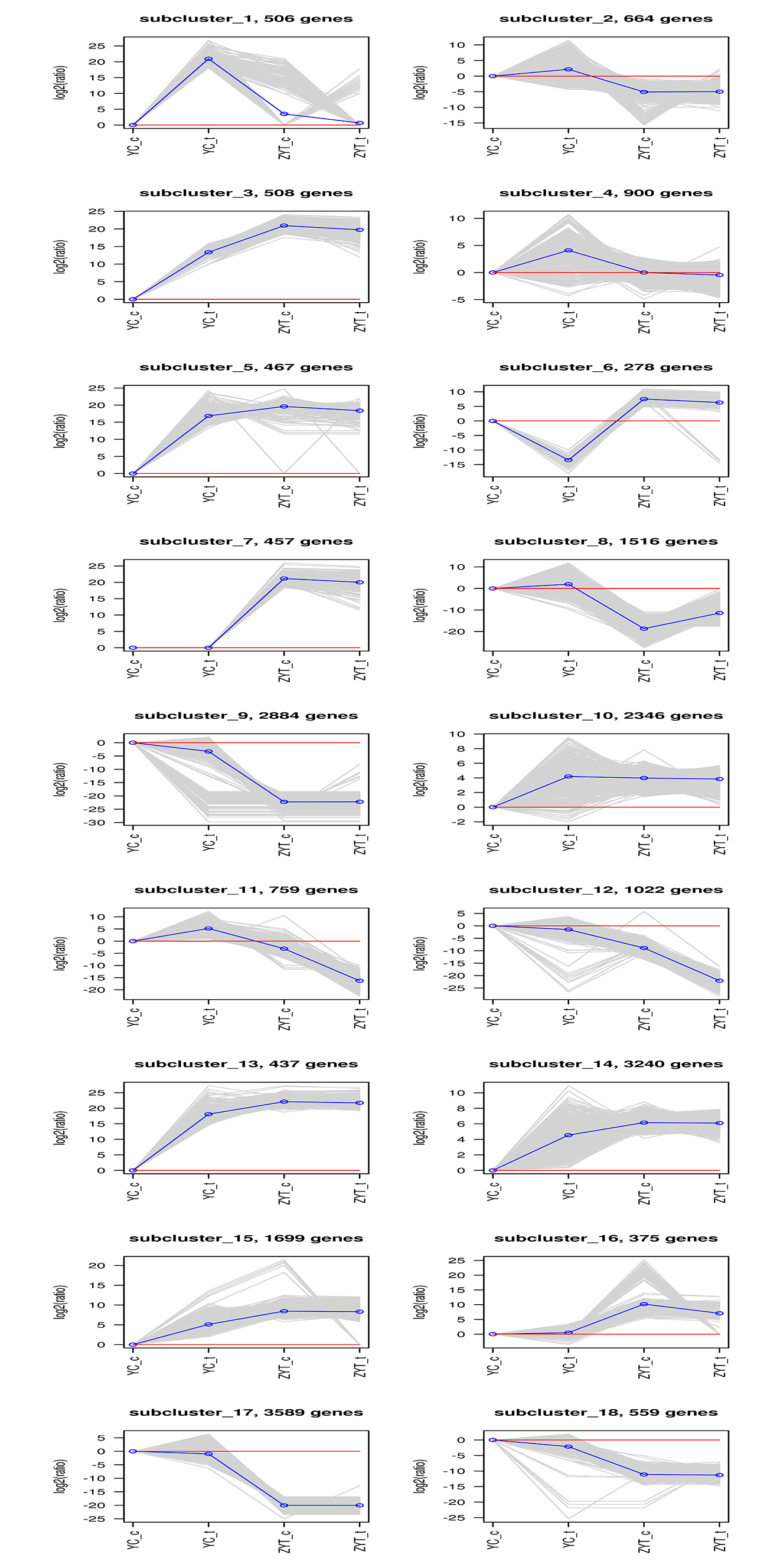

Supplement: Supplementary file 5 — Eighteen regulatory patterns determined by K-means clustering of all the DEGs expressed in theront and trophont groups of Cryptocaryon irritans with Lc-pis treatment. (JPEG 929 kb) [file 12864_2018_4565_MOESM5_ESM.jpg]

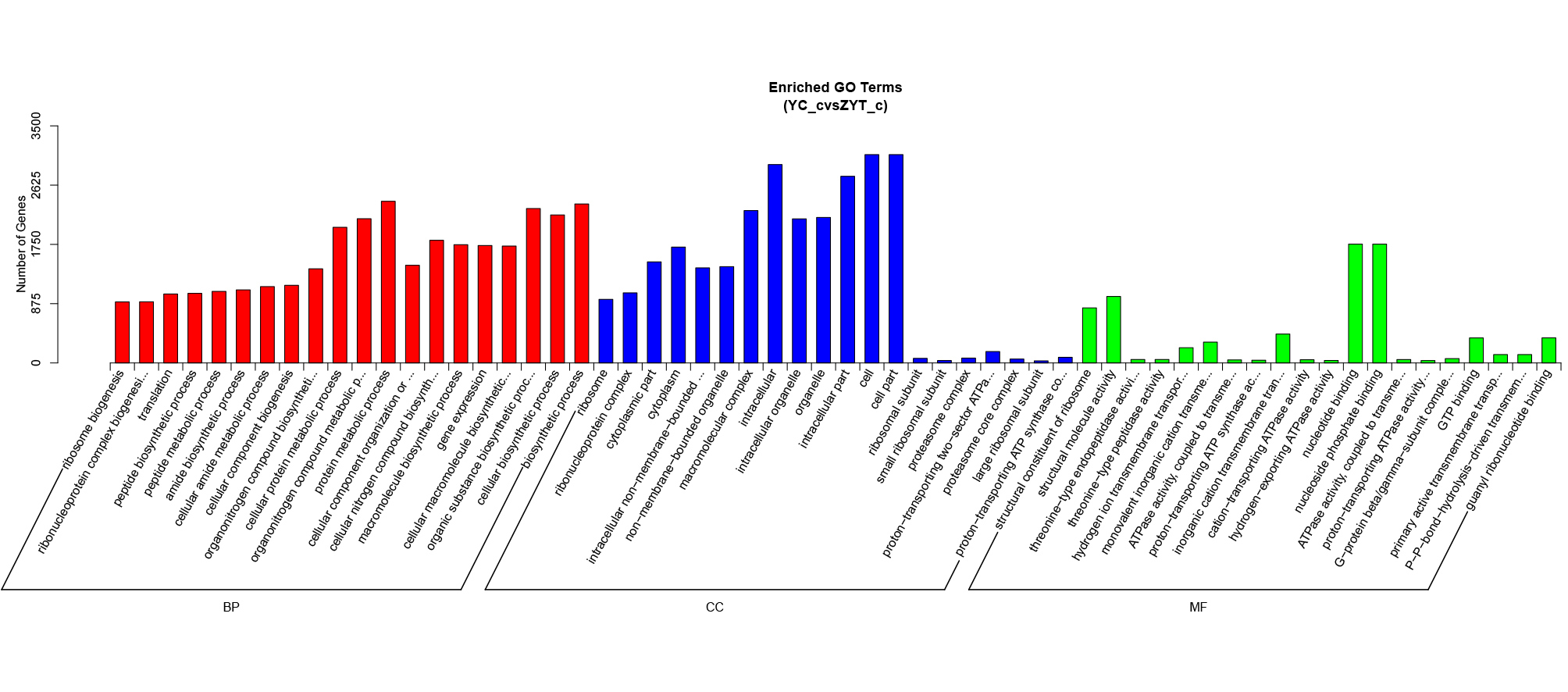

Supplement: Supplementary file 6 — GO enrichment bar graphs of DEGs between untreated theronts and untreated trophonts of Cryptocaryon irritans. (JPEG 504 kb) [file 12864_2018_4565_MOESM6_ESM.jpg]

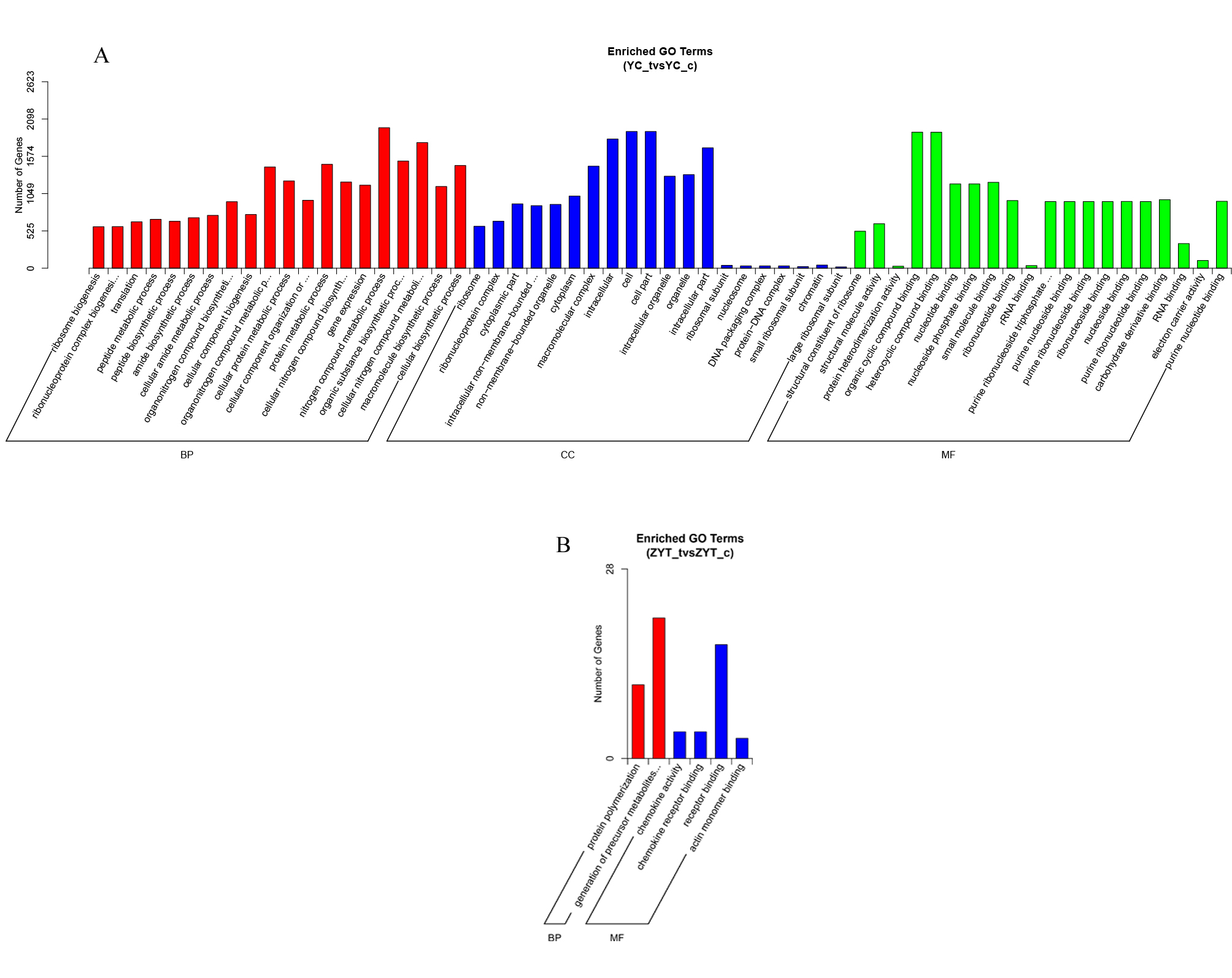

Supplement: Supplementary file 7 — GO classification of the DEGs. A: YC-t vs YC-c, B: ZYT-t vs ZYT-c. (JPEG 641 kb) [file 12864_2018_4565_MOESM7_ESM.jpg]

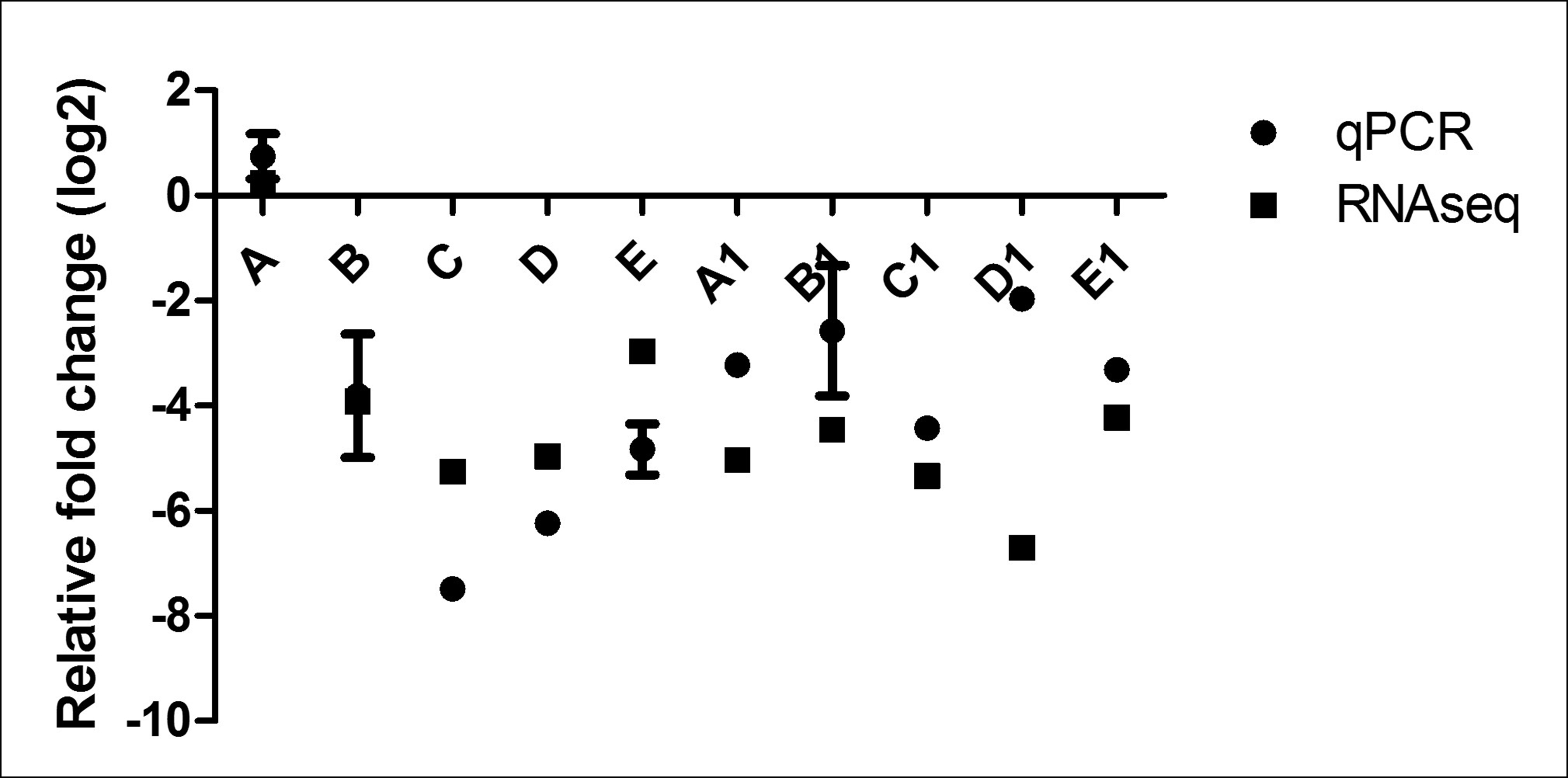

Supplement: Supplementary file 8 — Expression profiles of five genes from four developmental stages of Cryptocaryon irritans from RNA-Seq and RT-qPCR. Log2-fold changes from RNA-Seq analysis were highly correlated with log2-fold change values from RT-qPCR. A, B, C, D and E represented heat shock protein 90, GTP-binding protein, serine/threonine kinase, serum/glucocorticoid regulated kinase, Rab 5, respectively. Log2-fold changes are relative to YC c (A, B, C, D and E for ZYT-c/ YC-c; A1, B1, C1, D1 and E1 for vs YC-t/ YC-c). (JPEG 673 kb) [file 12864_2018_4565_MOESM8_ESM.jpg]
